# Ascariasis Associated with Pig Farming — Maine, 2010–2013

**Published:** 2013-05-24

**Authors:** Kate Colby, Stephen Sears, Elizabeth McEvoy, Don Hoenig, Blaine Mathison, Marcos de Almeida, Alexandre J. da Silva, Henry Bishop, Susan P. Montgomery, Susan Manning, Leigh Ann Miller

**Affiliations:** Maine Dept of Health and Human Svcs and Univ of Southern Maine; Maine Dept of Health and Human Svcs; Maine Dept of Agriculture, Conservation, and Forestry; Div of Parasitic Disease and Malaria, Center for Global Health; Career Epidemiology Field Officer Program; EIS Officer, CDC

During April 2010–March 2013, the Maine Department of Health and Human Services investigated multiple cases of ascariasis that had been reported by health-care providers, veterinarians, and patients. All of the cases were in persons who had lived or worked on Maine farms and had frequent exposure to pigs. Ascariasis, a parasitic roundworm infection caused by *Ascaris* species, is the most common human intestinal worm infection globally.* However, because ascariasis is not a reportable disease, limited data exist regarding the incidence of this infection in the United States (1), and the number of annual cases in Maine is unknown. After investigation, 14 persons on seven farms in Maine were identified with *Ascaris* infection.

To better assess the extent of the ascariasis problem, state health officials conducted field investigations at four of the seven farms with reported cases and collected worms from humans and pigs and from pooled pig feces. Human worm and pig worm specimens were sent to CDC for identification and analysis. Confirmed cases were among persons who had excreted in stool at least one worm laboratory-identified as *Ascaris* species. Probable cases were among persons who reported excreting at least one worm in stool and who were epidemiologically associated with a confirmed case. Suspected cases were among persons with symptoms consistent with larval migration (e.g., coughing up larvae) and who were epidemiologically associated with a confirmed case or who had excreted at least one worm in stool without laboratory confirmation or epidemiologic association with a confirmed case.

A total of 14 patients aged 1–53 years (median: 25 years) from seven farms in six Maine counties had an *Ascaris* infection (eight confirmed, four probable, and two suspected) during 2010–2013. Thirteen (93%) patients were female. Ten (71%) patients reported no international travel history; of the four patients with a history of international travel, two reported previous treatment for parasites, and two reported no previous screening or treatment. All patients sought medical care and were prescribed anthelminthic medication (e.g., albendazole).

Private reference and university laboratories confirmed *Ascaris* species in human samples from three farms and in pooled pig feces from two farms. CDC confirmed as *Ascaris* species four worms collected from humans at four different farms and worms collected from pigs at one of those farms. Transmission from pigs to humans has been reported in other countries and likely occurred on the seven farms in Maine (2). Occurrence of infections among persons with no other likely source of infection and common exposure to pigs suggests that pigs were the source of human infections.

Ascariasis is transmitted by the fecal-oral route. *Ascaris* eggs and adult worms are excreted in stool. *Ascaris* infections often are asymptomatic among humans, but symptoms can include gastrointestinal discomfort and cough. Adverse health outcomes can include lung inflammation, intestinal obstruction, and growth delays.

The seven implicated farms grew either organic or conventional produce and raised livestock for household consumption and/or local sale. This unusual disease cluster holds implications for limited-scale agriculture with respect to farming practices and concern over foodborne transmission. Investigators recorded field notes from each of the four farm visits and conducted case investigation interviews regarding international travel history, farming practices, animal husbandry, and hand hygiene. Recommendations to prevent human illness at farms where *Ascaris* infection has been confirmed include improved hand hygiene, growing vegetables away from areas where pigs are penned, discontinuing use of pig manure as fertilizer, and thoroughly washing produce.

## Footnotes

* Additional information available at http://www.cdc.gov/parasites/ascariasis/index.html.
